# A systematic review of peer support interventions to improve psychosocial functioning among cancer survivors: can findings be translated to survivors with a rare cancer living rurally?

**DOI:** 10.1186/s13023-024-03477-3

**Published:** 2024-12-20

**Authors:** L. Hemming, S. F. A. Duijts, N. Zomerdijk, C. Cockburn, E. Yuen, R. Hardman, J. Van Vuuren, T. Farrugia, C. Wilson, E. Spelten

**Affiliations:** 1 Violet Vines Marshman Centre for Rural Health Research, Rural Health School, La Trobe University, Bendigo, VIC 3552 Australia; 2 Department of Research and Development, Netherlands Comprehensive Cancer Organisation (Integraal Kankercentrum Nederland, IKNL), Utrecht, The Netherlands; 3 Department of Medical Psychology, Amsterdam University Medical Center, Location Vrije Universiteit, Amsterdam, The Netherlands; 4 Cancer Center Amsterdam, Cancer Treatment and Quality of Life, Amsterdam, The Netherlands; 5 Melbourne School of Psychological Sciences, University of Melbourne, Melbourne, VIC Australia; 6 Rare Cancers Australia, Bowral, NSW Australia; 7 School of Nursing and Midwifery, Deakin University, Geelong, VIC Australia; 8 Centre for Quality and Patient Safety Research, Institute for Health Transformation, Deakin University, Geelong, VIC Australia; 9 Centre for Quality and Patient Safety Research – Monash Health Partnership, Monash Health, Clayton, VIC Australia; 10 Sunraysia Community Health Services, Mildura, VIC Australia; 11 Melbourne School of Population and Global Health, Melbourne University, Melbourne, VIC Australia

**Keywords:** Cancer, Rare cancer, Psychosocial functioning, Peer support, Digital health, Rural, Regional and remote health

## Abstract

**Background:**

This study aimed to (1) summarise research on the impact of peer support interventions aimed at improving psychosocial functioning among cancer survivors, and (2) identify key components for developing a support intervention for patients with a rare cancer living in rural, regional or remote areas.

**Methods:**

A comprehensive search of EMBASE, MEDLINE, PsycINFO, CINAHL, and the Cochrane Library identified papers that examined peer support interventions: (i) for rare cancer patients, or (ii) for those living in rural, regional or remote locations, or (iii) that provided support online or via telehealth. After screening, data on study characteristics, intervention components and impact on psychosocial functioning were extracted. Quality assessment was conducted, and findings were synthesised narratively.

**Results:**

A total of 23 unique studies were included, primarily exploring peer support for middle-aged females with a breast cancer diagnosis. Interventions were online or telephone-based, targeting a range of psychosocial outcomes with significant improvements found for coping abilities and loneliness. The most impactful interventions involved online, group formats facilitated by healthcare professionals. There were limited data on rare cancers and rural populations.

**Conclusions:**

Few studies have explored peer support interventions for those diagnosed with a rare cancer living in rural, regional or remote areas. Evidence shows mixed impact on psychosocial functioning for cancer survivors, yet promising elements of peer support that can be translated to rare cancer patients living in rural, regional or remote areas.

**Supplementary Information:**

The online version contains supplementary material available at 10.1186/s13023-024-03477-3.

## Background

In 2020, there was an estimated 18.1 million cancer cases around the world [1], accounting for one in six deaths globally [2]. People diagnosed with cancer often experience adverse psychosocial outcomes [3]. For example, up to a third of cancer patients in acute care hospitals were found to have a diagnosed mental health disorder requiring treatment [4]. Cancer patients also report significant physical, emotional and social challenges which affect overall wellbeing and quality of life [5].

There is a growing appreciation of the need for community based, peer-to-peer support, rooted in patient activation and patient empowerment principles [6]. Peer support is defined as ‘a system of giving and receiving help founded on key principles of respect, shared responsibility, and an agreement of what is helpful’ [7, page 6]. Peer support can be provided one-on-one or through groups [6, 8]. Groups can be led by either trained professionals or by cancer patients, the latter with or without formal supervision and training in cancer support or group facilitation [6, 8]. Increasingly, owing in part to the covid-19 pandemic, peer support is delivered remotely, either via telephone or using online forums or videoconferencing software [9]. Peer support is considered particularly effective when programs are developed in collaboration with individuals who have lived experience, including cancer survivors and carers [10].

There is growing recognition of the importance of peer support [11]. Indeed, in several reviews the impact of peer support for cancer patients on a range of psychological outcomes has been demonstrated [6, 8, 11, 12, 51]. Findings have shown peer support to decrease anxiety, depression, social isolation and stigma, and increase understanding of cancer-related information, hope and optimism, psychological empowerment, stress management skills and quality of life [12]. Additionally, high workloads for healthcare professionals, as well as a recent shift towards patient empowerment, has led to an increased interest in supporting self-management of psychosocial outcomes by cancer patients [6].

Despite a large volume of research exploring the benefits of peer support for cancer patients in general, there is little research that focuses specifically on the benefits of peer support for those diagnosed with a rare form of cancer. Rare cancers are defined as those with an incidence rate of less than 6 cases in 100,000 people per annum [13]. Despite the label ‘rare’, one in five cancer patients is diagnosed with a rare tumour type, and more than 85% of all identified tumour types can be considered rare [14]. Patients diagnosed with a rare cancer face a more challenging illness trajectory than those with a common cancer, including delays in diagnosis, receiving incorrect treatment, and having limited access to clinical trials [15]. Moreover, the 5-year survival rate for individuals with a rare cancer is notably lower at 52% compared to 69% for those with a common cancer [16]. Recent evidence suggests that rare cancer patients also experience worse psychosocial outcomes than those with a common cancer, including higher prevalence of suicide and PTSD [17].

In addition to the significant challenges rare cancer patients deal with following their diagnosis, those living in regions far from the main population centres are challenged in accessing clinical, psychological, and informational support. There is little research exploring the impact of peer support on psychosocial functioning for cancer patients living in rural, regional or remote areas. Recent data has indicated that cancer patients living further away from metropolitan centres are at a higher risk of dying within five years of diagnosis [18]. Like rare cancer patients, rural cancer patients encounter numerous challenges in accessing care such as; limited availability of treatments and support providers, transportation barriers, financial challenges, and restricted access to clinical trials [19].

Those diagnosed with a rare cancer and who live in a rural, regional or remote area are therefore doubly challenged, yet there is a lack of evidence regarding how support can best be tailored to meet their needs. Therefore, the aims of the present study were to: (1) summarise research on the impact of peer support interventions aimed at improving psychosocial functioning among cancer survivors, and (2) identify key components for inclusion in a support intervention for patients with a rare cancer living in rural, regional or remote areas. By addressing these gaps, healthcare providers and policymakers can improve the quality of life and overall outcomes for patients with a rare cancer living in rural, regional, or remote areas.

## Methods

### Search strategy

This review adheres to the Preferred Reporting Items for Systematic Reviews and Meta-Analyses (PRISMA) guidelines [20]. Eligible studies were identified through comprehensive searches conducted in the following databases: EMBASE, MEDLINE, PsycINFO, CINAHL, and the Cochrane Library. The searches were carried out in September 2023 and updated in February 2024. There were no restrictions placed on publication date.

The search strategy was structured following the PICO format (Population/Intervention/Comparison/Outcome). The population of interest encompassed individuals of all age groups diagnosed with cancer. The primary intervention of interest was peer support, and the key outcomes were assorted measures of quality of life and psychosocial functioning. The search strategy was collaboratively developed in conjunction with a librarian consultant and incorporated terms related to the following key concepts: cancer AND peer support AND psychosocial outcomes AND (rare OR rural OR online). For a detailed overview of the complete search strategy, please refer to Supplement 1.

### Eligibility criteria

Studies were eligible for inclusion if: (1) they were published in peer-reviewed journals, encompassing any study design, and written in English; (2) the full text was accessible; (3) they included human participants of any age diagnosed with any type of cancer; (4) they incorporated an intervention utilising some form of peer support for cancer patients, with at least one of the following three characteristics: patients were diagnosed with a rare cancer, patients lived in rural, regional or remote areas, or the intervention was conducted remotely, and (5) the study reported at least one psychosocial outcome (e.g., quality of life, well-being, anxiety, depression, loneliness, isolation, or other mental health issue).

Studies were excluded from the review if: (1) it was not possible to segregate data related to cancer patients from data involving other participants, or where less than 30% of participants had a cancer diagnosis (a figure deemed appropriate by the research team to allow sufficient focus on cancer), (2) it did not focus on rural, regional, or remote patients—the intervention was not conducted remotely, and it was not possible to segregate data specific to rare cancer patients from other cancer patients, with at least 30% of participants having a rare cancer diagnosis (as above, a figure deemed by the research team to indicate sufficient focus on rare cancers) (3) the peer support program comprised a physical intervention (even where psychosocial outcomes were reported) (4) no original findings were reported (e.g., reviews, book chapters, clinical guidelines, letters, study protocols, editorials, erratum/corrections, obituaries), and/or (5) no peer review was performed (e.g., conference/meeting abstracts, dissertations and theses).

The definition of peer support was broad, encompassing diverse types and formats, including both synchronous and asynchronous support, peer education programs, and support groups facilitated by professionals with the aim of promoting peer support among cancer patients. Remote peer support interventions included online meetings, telehealth, mobile health (mHealth), mobile phone applications, and online discussion forums. The classification of rare cancers adhered to the RARECARE definition [13], defining them as having an incidence rate of fewer than 6 cases per 100,000 persons per year.

### Study selection and risk of bias

The systematic review process incorporated use of ASReview, a machine learning tool designed for systematic reviews [21]. This tool requires users to label studies as relevant or irrelevant regarding a specific research question. These data train the tool to identify relevant papers. The initial screening paper phase involved the first author (LH) assessing 1,132 articles in ASReview. Building on a previous study on the efficacy of semi-automated screening tools, including ASReview [22], and guided by researchers’ input, a decision was made to exclude articles deemed irrelevant after 100 consecutive instances of non-eligibility. This process resulted in a total of 802 records deemed relevant. These were imported into the systematic review software Covidence, where all title and abstracts were screened by two authors (involving LH, NZ & SD). The full texts of potentially eligible articles were obtained and screened independently by two authors (involving LH, SD, ES, CW, TF & EY). Any disparities that arose between the authors at any stage of the selection process were resolved through discussion.

Due to the range of included study types in this review, a number of quality assessment tools were used; the Cochrane Risk of Bias tool [23], the Quality Assessment Tool for Before-After (Pre-Post) Studies With No Control Group [24], the CASP Qualitative Studies Checklist [25], and the JBI Critical Appraisal Checklist for Analytical Cross Sectional Studies [26]. Risk of bias assessment was conducted concomitantly with data extraction and was performed in duplicate by LH, RH, JVV & TF.

### Data extraction and synthesis

A data extraction template was created using Covidence and was piloted by LH. Data were extracted by LH, RH, JVV & TF, with all studies being checked by a second author. Any conflicts were resolved through discussion. Data extraction involved recording information pertaining to study details (e.g., authors, year, country), methods (e.g., study design, outcome measures), participants (e.g., number of participants, age, gender, education level, working status, ethnicity, relationship status), disease characteristics (e.g., diagnosis), intervention characteristics (e.g., mode, format, communication type, frequency, length, facilitator, patient or carer involvement in creation) and outcomes (e.g., quality of life, well-being, anxiety, depression, loneliness, isolation, or other mental health issues). A narrative synthesis was applied to included studies.

## Results

### Study characteristics and risk of bias

**Fig. 1:**
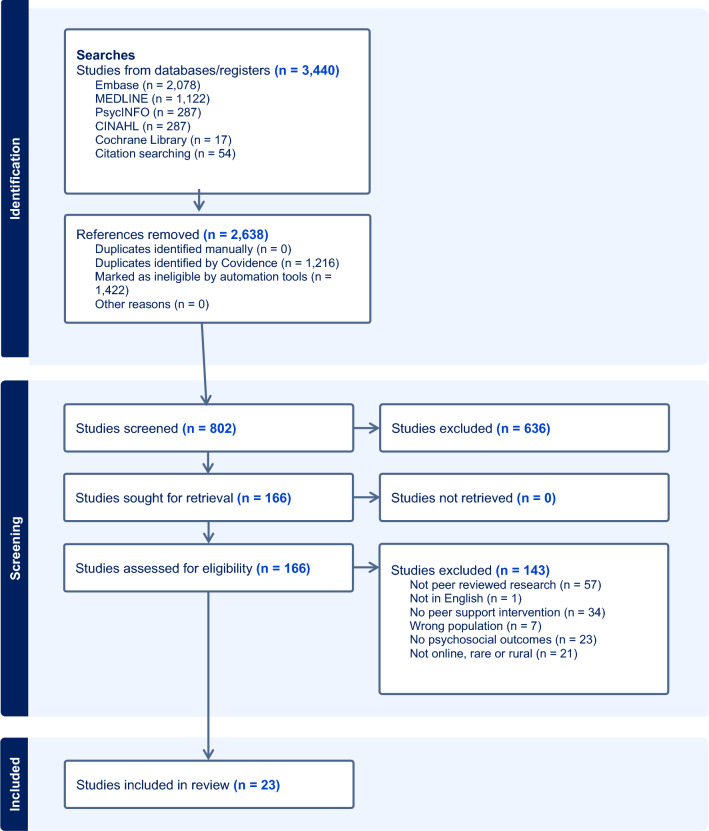
PRISMA flow diagram

Of the 802 identified records, 23 unique studies were included in the review based on full text screening. Data from two studies were merged together because they included the same sample, and therefore there are two references for one study included in the review [40, 41]. See Fig. 1 for the PRISMA flow chart of included studies and Table 1 for details of the studies included.

**Table 1:** Characteristics of included studies

References	Year	Country	Study design	N	Gender	Mean age	Diagnosis	Online, rare or rural?	Outcomes
Akers et al. [27]	2021	UK	Qualitative	11	100% male	67	Penile cancer	Rare cancers	Loneliness
Bender et al. [28]	2022	Canada	Quasi-experimental	34	Not reported	Not reported	Prostate cancer	Online or telephone	Anxiety, depression, quality of life, perceived social support
Canella et al. [29]	2023	Switzerland	Mixed methods	165	24% male	44.8	Mixed	Online or telephone. Included rare cancer patients	Coping
Changrani et al. [30]	2008	United States	Randomised controlled trial	68	0% male	46.8	Breast cancer	Online or telephone	Depression, quality of life, personal growth
Crane-Okada et al. [31]	2012	United States	Randomised controlled trial	142	0% male	61.8	Breast cancer	Online or telephone	Anxiety, coping, perceived social support
Gotay et al. [32]	2007	United States	Randomised controlled trial	305	0% male	Not reported	Breast cancer	Online or telephone	Depression, distress
Harmon et al. [33]	2021	United States	Cross-sectional observational	102	0% male	59.2	Breast cancer	Online or telephone	Anxiety, coping
Hirayama et al. [34]	2022	Japan	Cross-sectional observational	47	57% male	Not reported	Mixed	Online or telephone. Included rare cancer patients	Coping
Houts et al. [35]	1986	United States	Randomised controlled trial	32	0% male	49.8	Gynaecologic	Online or telephone. Included rare cancer patients	Depression, distress
Hoybye et al. [36]	2010	Denmark	Randomised controlled trial	794	11% male	54.1	Mixed	Online or telephone. Included rare cancer patients	Depression, distress
Kaal et al. [37]	2018	Netherlands	Cross-sectional observational	66	62% male	29.8	Mixed	Online or telephone. Included rare cancer patients	Loneliness
Klemm [38]	2012	United States	Longitudinal	50	0% male	52.2	Breast cancer	Online or telephone	Depression
Kosugi et al. [39]	2021	Japan	Cross-sectional observational	334	20% male	43.1	Mixed	Online or telephone. Included rare cancer patients	Loneliness
Lepore et al. [40, 41]^**1**^	2019	United States	Randomised controlled trial	183	0% male	52.3	Breast cancer	Online or telephone	Anxiety, depression, distress
Lieberman and Lepore [42]	2017	United States	Quasi-experimental	370	0% male	46.2	Breast cancer	Online or telephone	Depression
Osei et al. [43]	2013	United States	Randomised controlled trial	40	100% male	67.2	Prostate cancer	Online or telephone	Quality of life
Salzer et al. [44]	2010	United States	Randomised controlled trial	78	0% male	Not reported	Breast cancer	Online or telephone	Distress, quality of life
Sansom-Daly et al. [45]	2021	Australia	Randomised controlled trial	40	48% male	20.6	Mixed	Online or telephone Included 30% living in regional locations. Included rare cancer patients	Anxiety, coping, depression
Setoyama, Yamazaki and Namayama [46]	2011	Japan	Cross-sectional observational	220	0% male	Not reported	Breast cancer	Online or telephone	Anxiety, depression
Toija et al. [47]	2019	Finland	Randomised controlled trial	260	0% male	60	Breast cancer	Online or telephone	Quality of life
van Erp et al. [48]	2023	Netherlands	Quasi-experimental	10	40% male	25.1	Mixed	Online or telephone. Included rare cancer patients	Distress, quality of life
Vilhauer, McClintock and Matthews [49]	2010	United States	Randomised controlled trial	30	0% male	52.7	Breast cancer	Online or telephone	Depression, wellbeing
Winzelberg et al. [50]	2003	United States	Randomised controlled trial	72	0% male	49.5	Breast cancer	Online or telephone	Anxiety, depression, PTSD, stress

^1^Data merged from two separate papers with the same sample

Of the twenty-three studies included in this review, twelve were conducted in the United States [30–33, 35, 38, 40, 42–44, 49, 50], three in Japan [34, 39, 46], two in The Netherlands [37, 48] and one each in Australia [45], Canada [28], Denmark [36], Finland [47], Switzerland [29] and the UK [27]. Most (83%) were conducted from 2010, with 35% of studies conducted from 2020 indicating a rapid increase in scholarly attention to this topic.

The included studies comprised data from a total of 3,453 participants. Of the studies that reported gender (k = 22) and age (k = 18), overall, 89% of participants were women and the average age of participants was 40.8 years (SD = 12.7). The majority of the sample were married or partnered (58.7%, k = 13), participants were well educated, with 64.1% of the sample having completed some form of study beyond high school (k = 7), and around half of the sample (51%, k = 10) were employed. Of the seven studies which included details on participant ethnicity, an average of 91% of participants were Caucasian.

Nearly half of included studies (k = 12) focussed on women with breast cancer [30–33, 38, 40–42, 44, 46, 47, 49, 50]. Seven studies included participants with a range of cancer diagnoses [29, 34, 36, 37, 39, 45, 48]. Two studies focused on men with prostate cancer [28, 43]. One study focussed on gynaecologic cancers [35], and one on penile cancer [27]. The latter was the only paper that focused specifically on a rare cancer population, meeting the inclusion criteria for rare cancer [27]. Eight additional papers, which met eligibility criteria for online interventions, included data from rare cancer patients, but it was not possible to separate this data from that of other cancer patients [29, 34–37, 39, 45, 48].

No papers specifically focused on a rural population. Although no studies formally met the inclusion criteria for including rural, regional or remote participants, one study [45] reported that 30% of participants lived in inner or outer regional areas and another [49] reported that 16% of participants lived in a rural location. Nearly all studies (k = 22) focussed on online or telephone interventions [28–50].

Twelve studies utilised a randomised controlled trial design [30–32, 35, 36, 40, 41, 43–45, 47, 49, 50], and of these, two were assessed as low risk of bias, four as unsure and six as high risk of bias. Five studies used a cross-sectional observational design [33, 34, 37, 39, 46]. Risk of bias was considered high across these studies in areas such as sample details, objective measurements and a lack of consideration for confounding factors. Three studies used a quasi-experimental design (pre-post) [28, 42, 48] and were all assessed as ‘fair’ risk of bias. One study used a qualitative design [27] and was appraised as low risk of bias. One study used a mixed methods design and showed a high risk of bias in its quantitative components but low risk of bias in its qualitative components [29]. Finally, one study utilised a longitudinal design (38), which was appraised as high risk of bias.

Studies explored the impact of peer support interventions on a variety of psychosocial outcomes. The most common outcomes included depression (k = 12) [28, 30, 32, 35, 36, 38, 40–42, 45, 46, 49, 50], anxiety (k = 7) [28, 31, 33, 40, 41, 45, 46, 50], distress (k = 6) [32, 35, 36, 40, 41, 44, 48], quality of life (k = 6) [28, 30, 43, 44, 47, 48] and coping (k = 5) [29, 31, 33, 34, 45]. Fewer studies explored loneliness (k = 3) [27, 37, 39], perceived social support (k = 2) [28, 31], wellbeing (k = 1) [49], personal growth (k = 1) [30], PTSD (k = 1) [50] and stress (k = 1) [50].

### Intervention characteristics

The most common form of peer support (k = 11) was online forums [33, 36–39, 42–44, 46, 49, 50]. Online and/or in-person group meetings were used in six studies [27, 30, 34, 45, 48]. One study used a combination of online meetings and an online forum [40, 41]. Online and/or telephone one-to-one support were used in five studies [28, 31, 32, 35, 47]. One study explored online cancer survival stories [29] (See Table 2).

**Table 2:** Characteristics of interventions

References	Type of peer support	Mode of delivery	Format	Communication type	Frequency	Length of intervention	Facilitator	Lived experience involvement
Akers et al. [27]	In-person group meetings	In-person	Group	Synchronous	Monthly	1–2 years	Health professional (Nurse)	None
Bender et al. [28]	Online/telephone one-to-one support	In-person; Online; Telephone	One-to-one	Synchronous	Variable	1–3 months	Cancer survivor	Some
Canella et al. [29]	Online cancer survival stories	Online	One-to-one	Asynchronous	24-h availability	Not reported	No facilitator	Some
Changrani et al. [30]	Online group meetings	Online	Group	Synchronous	Weekly	30 weeks	Trained non-healthcare professional	Some
Crane-Okada et al. [31]	Telephone one-to-one support	Telephone	One-to-one	Synchronous	Weekly	7–12 months	Trained non-healthcare professional	None
Gotay et al. [32]	Telephone one-to-one support	Telephone	One-to-one	Synchronous	Weekly	4–8 sessions	Cancer survivor	None
Harmon et al. [33]	Online forum	Online	Group	Asynchronous	24-h availability	7–12 months	Trained non-healthcare professional	None
Hirayama et al. [34]	Online group meetings	Online	Group	Synchronous	Monthly	Not reported	Trained non-healthcare professional	None
Houts et al. [35]	Telephone one-to-one support	Telephone	One-to-one	Synchronous	Variable	3 calls	Cancer survivor	Some
Hoybye et al. [36]	Online forum	Online	Group	Synchronous	24-h availability	13 months	No facilitator	None
Kaal et al. [37]	Online forum	Online	Group	Asynchronous	24-h availability	Not reported	Trained non-healthcare professional	Some
Klemm [38]	Online forum	Online	Group	Asynchronous	24-h availability	12 weeks	Health professional (Social worker)	None
Kosugi et al. [39]	Online forum	Online	Group	Asynchronous	24-h availability	Not reported	Trained non-healthcare professional	None
Lepore et al. [40, 41]^1^	Online group meetings; online forum	Online	Group	Synchronous	Weekly	6 weeks	Health professional (Not specified)	None
Lieberman and Lepore [42]	Online forum	Online	Group	Asynchronous	Variable	6 months	Health professional (Not specified)	None
Osei et al. [43]	Online forum	Online	Group	Asynchronous	3 times a week	1–3 months	Not reported	None
Salzer et al. [44]	Online forum	Online	Group	Asynchronous	Variable	7–12 months	No facilitator	None
Sansom-Daly et al. [45]	Online group meetings	Online	Group	Asynchronous	Weekly	1–3 months	Health professional (Psychologist)	None
Setoyama, Yamazaki and Namayama [46]	Online forum	Online	Group	Asynchronous	Variable	Not reported	Not reported	None
Toija et al. [47]	Telephone one-to-one support	Telephone	One-to-one	Synchronous	Variable	Not reported	Cancer survivor	None
van Erp et al. [48]	Online group meetings	Online	Group	Synchronous	Weekly	6 weeks	Health professional (Psychologist)	Some
Vilhauer, McClintock and Matthews [49]	Online forum	Online	Group	Asynchronous	24-h availability	2 + years	No facilitator	None
Winzelberg et al. [50]	Online forum	Online	Group	Asynchronous	24-h availability	12 weeks	Health professional (Mental health practitioner)	None

^1^Data merged from two separate papers with the same sample

Seventeen of the interventions were delivered online [29, 30, 33, 34, 36–46, 48–50], four over the telephone [31, 32, 35, 47], one in person [27], and one had a blended format, with online, over the telephone and in-person delivery [28]. Seventeen interventions used a group format [27, 30, 33, 34, 36–46, 48–50] and six used a one-to-one format [28, 29, 31, 32, 35, 47]. Twelve interventions were asynchronous [29, 33, 37–39, 42–46, 49, 50] and eleven were synchronous [27, 28, 30–32, 34–36, 40, 41, 47, 48]. Of the synchronous interventions, five occurred weekly [30–32, 40, 41, 48], three occurred with a variable frequency [28, 35, 47] and two occurred monthly [27, 34]. Duration of interventions were: 1–3 months (k = 7) [28, 38, 40, 41, 43, 45, 48, 50], 4–6 months (k = 1) [42], 7–12 months (k = 4) [30, 31, 33, 44], 1–2 years (k = 2) [27, 36], 2 + years (k = 1) [49], or of a variable length (k = 3) [29, 32, 35]. The remaining five studies did not report the duration of the intervention [34, 37, 39, 46, 47]. Of the 21 studies where the facilitator was reported, 33% of interventions were facilitated by health professionals (e.g. nurse, social worker, psychologist) [27, 38, 40–42, 45, 48, 50], 29% were facilitated by trained non-professionals [30, 31, 33, 34, 37, 39], 19% were facilitated by cancer survivors [28, 32, 35, 47] and 19% had no facilitator [29, 36, 44, 49]. Only six studies (26%) reported involving individuals with lived experience in the design and development of the intervention [28–30, 35, 37, 48].

Interventions offered topics for discussion as well as a more open agenda to be led by patients. Combining data across both, the most commonly discussed topics included relationships with others (k = 8), physical symptoms and side effects (k = 7), emotions (k = 4), information needs, sexuality, spirituality, the future, day to day life and existentialism (k = 3) and practical matters, medical team and self-esteem (k = 2).

### The impact of peer support interventions on psychosocial outcomes

This section reports on aggregated results across all research designs (randomised controlled trials, quasi-experimental designs, cross-sectional observational and qualitative studies).

#### Depression

Of the twelve studies that examined the impact of peer support on depression, mixed findings were reported. Six studies (50%) revealed no significant difference in depression symptoms following intervention [28, 30, 32, 35, 38, 49]. One study [36] found less improvement in depression in the intervention group than the control group 6 months post-intervention. Three studies reported a significant reduction in depression following the intervention [40–42, 50], while an additional study showed an improvement in depression symptoms, although significance was not reported [45]. Finally, one study [46] found that conflict in online forums was associated with an increase in depression scores, whilst emotional expression was associated with a decrease.

#### Anxiety

Seven studies explored the impact of peer support on anxiety. The majority of studies (k = 4) found that peer support had no significant impact on anxiety [28, 31, 45, 50], with one exception finding a significant decrease in anxiety post-intervention [40, 41]. One study found that 10% of peer support participants reported an increase in fear and anxiety [33]. In another study [46] using peer support forums for emotional support, advice and insight was associated with a reduction in anxiety.

#### Distress

In the six studies that examined the impact of peer support on distress, mixed findings were reported. Three studies (50%) showed no changes in distress [32, 35, 36]. Two studies [40, 41, 48] showed a significant improvement in distress, although one of these was measured immediately post-chat [40, 41]. Another study showed an improvement in distress, however the sample size was too small to conclude whether this was significant [44].

#### Quality of life

Six studies reported on the impact of peer support on quality of life. The majority of studies (k = 5) found no statistically significant impact [28, 30, 43, 47, 48]. One study [44] found a decreased quality of life in the intervention compared to the control group, though this did not reach statistical significance.

#### Coping

Six studies explored the impact of peer support on coping with cancer, suggesting a positive impact. Three studies found that participants agreed that the peer support intervention had improved their ability to cope with cancer [29, 33, 34]. Two studies found that those who had received an intervention were more likely to use positive coping strategies following the intervention than those who had received care as usual [31, 45]. Finally, in one study [49], those in the control group showed a decline in ability to cope with breast cancer, which led to a significant difference in coping between intervention and control group at follow-up.

#### Loneliness

There were largely positive findings for the impact of peer support on loneliness; one study [37] found that around half of participants in an online forum agreed ‘I do not feel lonely anymore’, ‘I have good contact with peers’ and ‘I make new friends’. Another reported that men who attended peer support group meetings spoke about enjoying the opportunity to make friends and share cancer-related experiences [27]. Conversely, one study found that weekly active users of an online peer support group were equally as likely to be classified as belonging to a ‘high loneliness group’ and a ‘low loneliness group’ [39].

#### Perceived social support

Two studies [28, 31] explored the impact of peer support on social support. Neither found that peer support had a significant impact on perceived social support.

#### Wellbeing, personal growth, PTSD and stress

Three studies examined the impact of peer support interventions on wellbeing, personal growth, or PTSD and stress. One study [49] found no differences in wellbeing scores between intervention and control group participants at a one month and two month follow-up. In another controlled study [30], non-significant findings were also reported on the impact of peer support on personal growth. However, in this study, those who had received the intervention showed improvements in seeing new possibilities and increased feelings of strength. In a study which examined the impact of peer support on PTSD and stress [50], those who used online forums showed a significant improvement in PTSD symptoms and a reduction in stress.

### The most impactful components of peer support

Of the included interventions, thirteen resulted in improvements in at least one outcome [27, 29, 31, 33, 34, 37, 40–42, 44, 45, 48–50]. Narrative synthesis identified common features of the most successful interventions, which were defined as those that either showed improvements in multiple outcomes or had only significant improvements without any null findings. This resulted in eight interventions [27, 29, 34, 37, 40–42, 45, 50]. The majority (k = 7) of these interventions were delivered online [29, 34, 37, 40–42, 45, 50] in a group format (k = 7) [27, 34, 37, 40–42, 45, 50] and were facilitated by a healthcare professional (k = 5) [27, 40–42, 45, 50]. The interventions were varied in terms of their frequency, communication type and length of intervention.

Nine studies did not find peer support to have a significant impact on any psychosocial outcomes [28, 30, 32, 35, 36, 38, 39, 43, 47]. Although these interventions were varied in their characteristics, some common features emerged. Nearly half (k = 4) were delivered via telephone in a one-to-one format and were facilitated by individuals with lived experience [28, 32, 35, 47].

### Peer support for rare cancer patients

In the only study focussed exclusively on rare cancer patients [27], the impact of an in-person monthly peer support group meeting was evaluated qualitatively. Six themes were identified: developing friendships, peer support, sharing experiences, support from the clinical team, receiving information and raising awareness. Overall, participants reported that peer support was helpful in decreasing loneliness in men in this penile cancer patient’s cohort.

In eight studies [29, 34–37, 39, 45, 48], patients with a rare cancer were included in the sample, although it was not possible to separate their results from those of common cancer patients. Across the full sample, peer support was found to have a positive impact on depression [45], distress [48], coping [29, 34, 45] and loneliness [37].

### Peer support for patients living in rural, regional or remote areas

Two studies reported the rurality of their participants. In these studies, only 30% [45] and 16% [49] of participants lived in rural, regional or remote areas. Both studies evaluated the impact of online peer support groups, though one focused on breast cancer patients [49] and the other focussed on young adult cancer survivors with various diagnoses [45]. The breast cancer peer support program used asynchronous online forums [49], while the program for young adult cancer survivors involved six weekly synchronous videoconference group sessions [45].. The study on young adult cancer survivors did not report any location-specific findings. However, qualitative findings from the breast cancer study indicated that online provision of the peer support intervention was helpful because it made groups easily accessible for those who lived in rural areas [49].

Additionally, one study, which did not report participants rurality, included a qualitative finding relevant to participant location. In contrast to the breast cancer findings, the study on men diagnosed with rare penile cancer revealed that participants were willing to travel up to four hours to attend in-person support groups.

## Discussion

### Main findings

In this review, peer support interventions aimed at improving psychosocial functioning among cancer survivors were explored to identify key components for inclusion in a peer support intervention suitable for patients with a rare cancer living in rural, regional or remote areas. A total of 23 unique studies were included in the review. Included interventions comprised of online forums, group meetings and one-to-one support. Of the included interventions, thirteen resulted in improvements in at least one outcome, with the most significant outcomes being coping and loneliness. The majority of these interventions were delivered online, in a group format, and were facilitated by a healthcare professional. Finally, there is not enough data available to report conclusively on the evidence for peer support for rare cancer survivors, living in rural, regional or remote areas.

### Interpretation of findings

In this review, peer support services fell into one of three categories: online forums, group meetings, and one-to-one support. Facilitators included health professionals, trained non-professionals, and cancer survivors, aligning with existing typologies of peer support based on delivery mode and facilitator type [8, 51]. Few interventions involved individuals with lived experience in their design, despite its importance for ensuring relevancy, acceptability and effectiveness [10]. This contradicts complex intervention development frameworks that emphasise involving individuals with lived experience [52].

The impact of peer support services on psychosocial outcomes was mixed, with improvements mainly identified in coping and loneliness. Previous reviews also found mixed results for the impact of peer support programs on psychosocial outcomes for cancer patients [51]. These mixed findings may be due to the heterogeneity of peer support interventions and the varied outcomes tested in the included studies, which make it difficult to compare results [53].

Online support groups facilitated by a healthcare professional led to the most improved psychosocial outcomes, while one-to-one telephone support with a cancer survivor had the least favourable impact. These findings partially support previous studies that found one-to-one face-to-face and group internet peer support groups to be most effective [51] but contradict others suggesting one-to-one face-to-face interventions are beneficial [12]. In one study, more than half of prostate cancer patients preferred face-to-face peer connections over internet-based ones [54]. Still, other studies suggest no differences in outcomes between face-to-face and online peer support groups, though user profiles may differ [55].

Research on peer support for rare cancer patients is limited. This review found that peer support was typically accessed by well-educated, middle-aged white females diagnosed with breast cancer. This supports previous research which has found this group benefits most frequently from peer support [51]. Indeed, economic analysis indicates more funding is invested into research and treatment of breast cancer than other cancer types [56]. Notably, the one study which examined the impact of a peer support program for people with rare cancer found several qualitative improvements for men with penile cancer [27]. Together, the findings suggest the need for further research and resources invested into examining the utility of peer support for broader groups of people diagnosed with cancer, including men, and those with rare cancer diagnoses.

This review highlights the lack of research involving cancer survivors living in rural, regional, and remote areas. Only two of the included studies reported on the rurality of participants, and none specifically met the inclusion criteria for targeting rural, regional, and remote populations. The findings suggested that online peer support can improve accessibility for those living remotely, though qualitative evidence indicated that participants are still willing to travel for in-person sessions. Despite limited research on psychosocial interventions for non-metropolitan areas, studies not specifically addressing geographic impact are still valuable. Research shows rural and urban cancer patients have similar psychosocial needs [57], suggesting these findings may generalise to this group. Additionally, studies on peer support for other rare diseases, focussing on sharing clinical information and personal experiences, have shown positive outcomes [58], suggesting peer support may also have an impact for rare cancer patients. This review includes peer support programs primarily delivered online or via telehealth, formats beneficial for patients with less common cancers [59], and those in geographically distant locations [60].

### Strengths and limitations

This study adds to our understanding of the impact of peer support on cancer patients. Whilst previous reviews have explored this topic, the current review focusses only on peer support groups that were either a) delivered online b) targeted rare cancer patients or c) targeted rural, regional and remote cancer patients. The search strategy and study selection process had several strengths. Firstly, the review adhered to the PRISMA guidelines, ensuring transparency and methodological rigour. The use of the ASReview tool for initial screening helped streamline the study selection process. Duplicate screening of titles and abstracts, as well as resolving conflicts through discussion, ensured the reliability of study selection.

However, there were also limitations. Restricting the search to studies written in English may have introduced language bias. Additionally, the review did not include grey literature, which could have provided valuable insights. Further, the findings of this review may be constrained due to methodological flaws of the included studies; less than half of the included studies utilised a randomised controlled trial design, and many included studies were found to be at high risk of bias. Finally, the majority of included studies focussed on peer support provided to women with breast cancer. It has been suggested that peer support may have fewer profound impacts on this population due to the abundance of support that is already available for breast cancer patients, and the findings may therefore not extend to patients with other cancers, particularly less common or rare cancers [51].

### Implications for research and practice

This review found that peer support programs typically targeted well-educated, middle-aged white females with breast cancer. Future peer support programs should aim to reach a broader range of demographics including those diagnosed with a rare cancer, and those living in rural, regional and remote areas.. Additionally, there was a notable lack of involvement of people with lived experience (i.e., patients and their carers) in the design of these interventions. Future efforts should focus on co-designing peer support programs, ensuring that those with lived experience are involved to enhance the relevance and acceptability of these programs for the target users.

The findings suggest that online group programs facilitated by healthcare professionals may produce the best outcomes, although results were inconclusive. More research is needed to determine which type of peer support is most effective. Well-designed, rigorous trials with sufficient power to detect meaningful differences in important psychosocial outcomes are also required, ensuring that adverse events are examined. Finally, interventions tailored specifically for rare cancer patients living in rural, regional, and remote areas are needed to address the current lack of research and investment in this population..

### Conclusion

Peer support is primarily delivered online in a group setting to patients with breast cancer. Although evidence of the impact of peer support services on various psychosocial outcomes is mixed, coping abilities and loneliness appeared to improve with peer support. Peer support that is delivered by a healthcare professional in an online group setting may lead to more positive outcomes than one-to-one telephone support provided by individuals with lived experience. However, there is a need for more research to determine which type of peer support yields the best results; the question remains of what works, for whom and why. Importantly, the findings highlight the lack of research and investment in peer support for those diagnosed with rare cancers living in rural, regional, or remote locations. Given the significant challenges that this unique, yet large, population faces, it is crucial to develop an intervention that serves the needs of this group.

## Supplementary Information

Additional file 1 (DOCX 19 KB)

## Data Availability

Data sharing is not applicable to this article as no datasets were generated or analysed during the current study.
