# Supplementary material for: A systematic review of peer support interventions to improve psychosocial functioning among cancer survivors: can findings be translated to survivors with a rare cancer living rurally?
Source: Orphanet J Rare Dis. 2024 Dec 20;19:473. doi: 10.1186/s13023-024-03477-3 (PMC11662424; doi:10.1186/s13023-024-03477-3)
Supplement: Supplementary file 1 — Additional file 1 (DOCX 19 KB) [file 13023_2024_3477_MOESM1_ESM.docx]

## Supplement 1 - Search strategy

| **Concept 1 – cancer AND** | **Concept 2 – peer support AND** | **Concept 3 – psychosocial outcomes OR quality of life AND** | **(Concept 4 – online OR** | **Concept 5 – rural OR** | **Concept 5 – rare)** |
| --- | --- | --- | --- | --- | --- |
| Neoplasms (MeSH) | Peer group (MeSH) | Psychosocial Support Systems (MeSH) | Online social networking (MeSH) | Rural population (MeSH) | Rare |
| Cancer* | (peer adj2 (support* OR counsel* OR coach* OR led OR lead* OR mentor* OR peer* OR assist* OR group*)) | Psychosocial Intervention (MeSH) | Online systems(MeSH) | Rural health (MeSH) | Uncommon |
| Tumo?r* |  | psychosocial* or "psycho social*" | Online or “on line" | Rural health services (MeSH) | Less common |
| Neoplas* |  | "quality of life" (MeSH) | computers (MeSH) | Medically underserved area (MeSH) | Neglected |
| Malignan* |  | psychological well-being (MeSH) | Computer* or electronic | Rural* |  |
| benign |  | quality adj2 life | Internet OR “web based” | Regional* (see how this goes) |  |
| Carcinoma* |  | wellbeing or "well being" | Cell phone (MeSH) | Remote* |  |
| Oncolog* |  | needs | ((cell* OR mobile OR smart) ADJ1 (phone* OR device*)) | Nonurban OR “non urban” |  |
| Chemotherap* |  | Anxiety (MeSH) | “I pad” OR “I phone” OR android | Nonmetropolitan OR “non metropolitan” |  |
|  |  | anxiety or anxious* | Computers, handheld (MeSH) | (country AD3 (town* OR area* OR communit* OR locat*)) |  |
|  |  | Depression (MeSH) |  | Bush |  |
|  |  | depress* |  | “geographic* isolat*” |  |
|  |  | Loneliness (MeSH) |  |  |  |
|  |  | loneliness or isolat* |  |  |  |
|  |  | connect* or belong* |  |  |  |
|  |  | Emotional Adjustment (MeSH) |  |  |  |
|  |  | Emotional Regulation (MeSH) |  |  |  |
|  |  | Emotion* |  |  |  |
|  |  | Social Interaction (MeSH) |  |  |  |
|  |  | Social* |  |  |  |
|  |  | Mental Health (MeSH) |  |  |  |
|  |  | “mental health” |  |  |  |
|  |  | Spirituality (MeSH) |  |  |  |
|  |  | Spirit* |  |  |  |
